# Prognostic model for osteoarthritis combining imaging and clinical biomarkers

**DOI:** 10.3389/fmed.2026.1722232

**Published:** 2026-03-18

**Authors:** Longjie Yuan, Yu Liang, Bin Liang, Yi Zhao, Kewei Duan, Luobin Ding

**Affiliations:** 1 Department of Sports Medicine, The Third Hospital of Shijiazhuang, Shijiazhuang, China; 2 Department of Ultrasonography, The Fourth Hospital of Shijiazhuang, Shijiazhuang, China; 3 Department of Bone and Joint Surgery II, The Third Hospital of Shijiazhuang, Shijiazhuang, China

**Keywords:** gradient boosting, imaging features, osteoarthritis, random forest model, support vector machine

## Abstract

**Objective:**

This study aims to construct and validate a prognostic prediction model for knee osteoarthritis (OA) based on baseline characteristics, imaging manifestations and clinical indicators, in order to effectively assess the risk of adverse outcomes of individual patients, and to provide scientific basis for early identification of high-risk groups, formulation of precise intervention strategies and individualized management plans.

**Methods:**

This retrospective study enrolled 345 knee OA patients. The patients were randomly divided into a training set (*n* = 241) and a validation set (*n* = 104) in a 7:3 ratio. Imaging features and clinical indicators were collected for all patients. In the training set, univariate analysis, least absolute shrinkage and selection operator (LASSO) regression, and multivariate logistic regression were applied to identify independent predictors. Furthermore, three machine learning models—Random Forest (RF), Support Vector Machine (SVM), and Gradient Boosting Machine (GBM)—were constructed. The predictive performance of each model was evaluated using the area under the receiver operating characteristic curve (AUC), calibration curve and decision curve analysis, and the optimal model was selected for final prediction, with the importance of key predictive variables analyzed.

**Results:**

No significant differences were observed in baseline characteristics between the training and validation sets (*p* > 0.05). Univariate analysis showed that body mass index (BMI), medial joint space width (mJSW), total bone marrow lesion volume (TBLV), tibiofemoral angle (TFA), Western Ontario and McMaster Universities Osteoarthritis Index (WOMAC) function subscore, serum high-sensitivity C-reactive protein (hs-CRP), and urinary C-terminal telopeptide of type II collagen (uCTX-II) were associated with treatment failure (all *p* < 0.05). Multivariate Logistic regression identified BMI, TBLV, TFA, WOMAC function subscore, serum hs-CRP, and uCTX-II as independent risk factors for sustained clinical deterioration in knee OA patients (*p* < 0.05), while mJSW was an independent protective factor (*p* < 0.05). The RF model exhibited the highest AUC (0.910), significantly outperforming the SVM (0.885) and GBM (0.824), thus being selected as the optimal model.

**Conclusion:**

The RF model, constructed based on imaging features and clinical indicators, effectively predicts the risk of sustained clinical deterioration in knee OA patients, with BMI, TBLV, and serum uCTX-II serving as key predictive markers.

## Introduction

Knee osteoarthritis (OA) is a prevalent degenerative joint disorder characterized pathologically by articular cartilage degeneration, osteophyte formation, and synovial inflammation, with clinical manifestations predominantly including pain, stiffness, and functional impairment, significantly impacting patients’ quality of life and imposing a substantial socioeconomic burden (1, 2). Current clinical management primarily focuses on symptomatic relief and lacks disease-modifying interventions. Approximately 20–30% of patients exhibit significant radiographic and symptomatic progression over several years, often necessitating joint replacement, which poses major challenges in clinical management (3). Traditional prognostic assessments predominantly rely on the Kellgren-Lawrence (K-L) radiographic grading system and subjective symptom scores, which are limited by low sensitivity, subjectivity, and delayed predictive capacity, hindering individualized risk stratification (4).

Recent studies have demonstrated that OA progression was closely associated with multiple imaging and clinical indicators (5, 6), but most existing large-scale studies (e.g., the Osteoarthritis Initiative) mainly rely on single-type data or general populations without standardized intervention backgrounds, leading to limited clinical applicability of their predictive models. To address this gap, we integrated imaging features, clinical scales, and biochemical biomarkers, and targeted OA patients with standardized baseline treatment to develop a more comprehensive and clinically translatable prognostic model. Imaging features, including bone marrow lesions (BMLs), cartilage defects, and meniscal extrusion, have been established as robust predictors of rapid disease progression in numerous studies (5, 6). Among clinical parameters, higher body mass index (BMI), persistent severe pain, and functional impairment are significantly correlated with poor outcomes. Additionally, systemic and local metabolic activity reflected by elevated levels of inflammatory and metabolic biomarkers, such as serum high-sensitivity C-reactive protein (hs-CRP) and urinary C-terminal telopeptide of type II collagen (uCTX-II), may further enhance predictive accuracy. However, single indicators exhibit limited predictive value, with sensitivities typically below 65%. Machine learning (ML) approaches, capable of integrating multidimensional heterogeneous data, have demonstrated superior performance in disease prediction. This study aims to develop a prognostic prediction model for OA based on joint imaging features, clinical indicators, and biochemical markers using ML algorithms. This model will facilitate the early identification of high-risk progression cases and provide an objective basis for personalized intervention and therapeutic decision-making.

## Materials and methods

### Study population

A total of 345 knee OA patients diagnosed at our hospital’s Department of Bone and Joint Surgery II between December 2021 and December 2024 were retrospectively enrolled. The patients were randomly divided into a training set (*n* = 241) and a validation set (*n* = 104) in a 7:3 ratio.

Inclusion criteria:

1) Diagnosis confirmed per the Diagnosis and Treatment Guidelines for Osteoarthritis (7, 8).
2) Receipt of standardized baseline treatment [in accordance with the Chinese Osteoarthritis Diagnosis and Treatment Guidelines (2021)] (7), including non-pharmacological interventions such as weight management and joint protection education, and conventional drug therapy such as oral non-steroidal anti-inflammatory drugs and topical analgesics) for ≥3 months, with the same treatment regimen maintained during the 6-month follow-up period.
3) Availability of complete baseline clinical data and scheduled follow-up records, including imaging examinations and efficacy assessments.
4) Complete imaging and serum inflammatory marker data.

Exclusion criteria:

1) Comorbidities with other joint diseases (e.g., rheumatoid arthritis, gouty arthritis, post-traumatic arthritis).
2) Severe cardiac, hepatic, or renal dysfunction or systemic major illnesses.
3) Active malignancies, acute infections, or inflammatory diseases.
4) Loss to follow-up or missing key clinical/imaging data during the study period.

This study was approved by the Ethics Committee of The Third Hospital of Shijiazhuang (No. LYLS2021-07-04), and written informed consent was obtained from all patients.

### Data collection

Clinical information collected included:

Demographics: Age, gender, BMI, symptom duration, prior intra-articular injection frequency.

Scale scores: (1) Western Ontario and McMaster Universities Osteoarthritis Index (WOMAC): Includes 24 items (5 for pain, 2 for stiffness, 17 for function), total score range 0–96, higher score indicates more severe OA symptoms; (2) Visual Analogue Scale (VAS): Score range 0–100 mm, higher score indicates more severe pain; (3) Knee injury and Osteoarthritis Outcome Score (KOOS): 42 items, total score 0–100, higher score indicates better knee function; (4) 36-Item Short Form Health Survey (SF-36): 36 items, total score 0–100, higher score indicates better health-related quality of life; (5) Hospital Anxiety and Depression Scale (HADS): 14 items (7 for anxiety, 7 for depression), each item score 0–3, total score 0–21, higher score indicates more severe anxiety/depression; (6) Timed Up and Go Test (TUG): Measures time (seconds) to complete the test, shorter time indicates better mobility; (7) 30-s Chair Stand Test (30s-CST): Number of repetitions in 30 s, more repetitions indicate better lower limb muscle strength; (8) 6-min walk distance: Distance (meters) walked in 6 min, longer distance indicates better exercise capacity; (9) Active range of flexion: Range of motion (degrees), larger range indicates better joint mobility; and (10) Extension deficit: Degrees of inability to fully extend the joint, larger value indicates worse joint function.

Biomarkers: Medial joint space width (mJSW), lateral joint space width (lJSW), maximum osteophyte height (MOH), total bone marrow lesion volume (TBLV), femoral medial condyle cartilage thickness (FMCCT), subchondral bone plate thickness (SBPT), medial meniscus extrusion distance (MMED), tibiofemoral angle (TFA), hs-CRP, and uCTX-II.

Measurement methods and reliability: (1) mJSW and lJSW were measured manually using ImageJ software (version 1.8.0) by two independent radiologists. Inter-rater and intra-rater reliability were evaluated using intraclass correlation coefficient (ICC), with ICC > 0.85 for both indicating good reliability. (2) TBLV was segmented from T2-weighted MRI images using ITK-SNAP software (version 3.8.0), and manual correction was performed by an experienced orthopedic surgeon to ensure accuracy. (3) The 6-min walk distance (6MWD) was conducted in accordance with the American Thoracic Society (ATS) guidelines: a 40-meter straight track was used, standardized verbal instructions were provided to patients, and rest was allowed if necessary.

### Outcome definition

Based on the Osteoarthritis Research Society International (OARSI) Treatment Response Criteria (2020) and the Chinese Osteoarthritis Diagnosis and Treatment Guidelines (2021), patients were stratified into two groups (9, 10):

Favorable prognosis group: Met all criteria during the 6-month follow-up: (1) Symptom stability: VAS change <20 mm and WOMAC functional subscore deterioration <20%. (2) Functional maintenance: 6-min walk distance decline <50 m. (3) Non-progressive imaging: No K-L grade increase and medial JSW reduction ≤0.5 mm. (4) No surgical intervention: No total knee arthroplasty (TKA).

Poor prognosis group (disease progression): Met any of the following: (1) Symptom/functional decline: VAS increase ≥20 mm and/or WOMAC functional subscore deterioration ≥40%. (2) Imaging progression: K-L grade increase ≥1 or mJSW reduction ≥0.5 mm. (3) Surgical requirement: TKA for end-stage OA.

### Sample size sufficiency

Sample size sufficiency was verified based on the criterion that at least 10–15 events (EPVs) were required for each predictor variable. 93 outcome events were observed (event rate, 27%), and seven predictive variables were finally included in the model, resulting in an EPV of 13.3, which met this criterion., In addition, a *post hoc* power analysis conducted using G*Power 3.1 indicated that with the current sample size, the statistical power to detect a clinically significant association (e.g., an odds ratio of 2.0) between key predictors and outcomes exceeded 80% (*α* = 0.05). In summary, these evaluations confirm that the sample size is adequate to support the reliability of model development and the validity of the research findings.

### Statistical analysis

Data were analyzed using SPSS 26.0, Python 3.8.5 and R 4.2.3. Normally distributed continuous variables were expressed as mean ± standard deviation (x̄ ± SD) and compared via independent *t*-tests; non-normally distributed variables were presented as median (interquartile range) and assessed via Mann–Whitney U test. Categorical data were described as *n* (%) and analyzed using χ^2^ tests. For data preprocessing, z-score normalization was performed for all continuous variables to eliminate the influence of different measurement units. In the training set, the potential variables were screened by univariate analysis. Variables with *p* < 0.05 in univariate analysis underwent least absolute shrinkage and selection operator (LASSO) regression for feature selection (lambda.1se criterion). The optimal tuning parameter was determined via 10-fold cross-validation to identify core predictive variables. Finally, multivariate Logistic regression was applied to identify independent predictors, and calculating odds ratios (OR) and their 95% confidence intervals (CI). Variance inflation factors (VIF) were calculated to exclude multicollinearity (VIF threshold <10).

Prior to final model construction, we initially tested advanced models including eXtreme gradient boosting, gradient boosting machine (GBM), random forest (RF), support vector machine (SVM), and neural networks. Subsequently, we constructed RF, SVM, and GBM using Python 3.8.5 and scikit-learn, with 10-fold cross-validation for hyperparameter optimization. Overfitting was evaluated by two methods: (1) comparing the AUC values of the training set and validation set (a small difference indicates low overfitting); (2) calculating the out-of-bag (OOB) error for the RF model (OOB error <0.2 indicates good generalization ability). Detailed hyperparameter tuning strategies: For RF, the grid search range included number of trees (100–500), maximum depth (3–10), and splitting criterion (Gini impurity/entropy); for SVM, the grid search range included kernel type (linear/RBF/polynomial), C (0.1–10), and gamma (0.01–1); for GBM, the grid search range included learning rate (0.01–0.2), number of estimators (100–300), and maximum depth (3–8). Model discrimination was evaluated via receiver operating characteristic (ROC) curve analysis, and the area under the curve (AUC) value was calculated. The bootstrap optimism correction was used to assess overfitting. A calibration curve was plotted and evaluated using the Hosmer-Lemeshow goodness-of-fit test. Decision curve analysis (DCA) was used to evaluate the clinical application value of the nomogram by calculating the net benefit at different threshold probabilities. The model was interpreted using SHapley Additive exPlanations (SHAP) values to quantify the contribution of each feature to the prediction outcome, and feature importance plots and dependence plots were generated. A nomogram model was developed based on the optimal model. A *p* value < 0.05 was considered statistically significant.

## Results

### Baseline characteristics of training and validation sets

The training set (*n* = 241) included 176 (73.03%) favorable and 65 (26.97%) poor prognosis cases, while the validation set (*n* = 104) comprised 76 (73.08%) and 28 (26.92%), respectively. No significant differences were observed between the two cohorts (*p* > 0.05) (Table 1).

**Table 1:** Comparison of baseline characteristics between training and validation sets.

Variables	Training set (*n* = 241)	Validation set (*n* = 104)	*t/χ^2^*	*P*
Age (years)	63.92 ± 7.13	64.31 ± 6.84	0.472	0.637
Gender (male/female)	110/131	49/55	0.063	0.801
BMI (kg/m^2^)	27.80 ± 3.50	28.40 ± 4.00	0.651	0.516
Symptom duration (months)	42.10 ± 19.50	40.32 ± 18.14	0.794	0.428
Prior injection frequency (times/year)	1.30 ± 0.70	1.20 ± 0.60	1.269	0.205
mJSW (mm)	3.08 ± 0.85	3.01 ± 0.80	0.714	0.476
IJSW (mm)	5.18 ± 1.10	5.27 ± 1.07	0.703	0.483
MOH (mm)	2.70 ± 0.73	2.63 ± 0.70	0.827	0.409
TBLV (cm^3^)	1.88 ± 0.97	1.80 ± 0.92	0.714	0.476
FMCCT (mm)	2.23 ± 0.50	2.28 ± 0.49	0.858	0.392
SBPT (mm)	3.60 ± 0.86	3.53 ± 0.83	0.701	0.484
MMED (mm)	3.95 ± 1.18	4.02 ± 1.15	0.510	0.610
TFA (degrees)	176.80 ± 3.50	177.20 ± 3.30	0.991	0.323
Pain VAS score (0–100 mm)	57.20 ± 14.30	55.60 ± 13.90	0.962	0.337
WOMAC pain subscore (0–20)	11.90 ± 3.30	11.50 ± 3.20	1.043	0.298
WOMAC function subscore (0–68)	40.10 ± 10.50	38.90 ± 10.00	0.988	0.324
6-minute walk distance (meters)	359.71 ± 75.82	366.44 ± 72.59	0.766	0.444
Active flexion angle (degrees)	121.87 ± 11.68	123.11 ± 11.08	0.912	0.359
Active extension lag (degrees)	2.90 ± 1.60	2.70 ± 1.50	1.085	0.279
Serum hs-CRP (mg/L)	3.95 ± 2.10	3.78 ± 1.99	0.701	0.484
uCTX-II (ng/mmol Cr)	385.40 ± 142.60	371.80 ± 136.90	0.823	0.411
30-second chair stand test (repetitions)	12.50 ± 3.80	12.90 ± 3.60	0.911	0.363

### Univariate analysis of factors associated with sustained clinical deterioration in knee OA patients

In the training set, univariate analysis demonstrated statistically significant differences between the favorable prognosis group and poor prognosis group regarding BMI, mJSW, TBLV, TFA, WOMAC function subscale scores, serum hs-CRP, and uCTX-II levels (all *p* < 0.05) (Table 2).

**Table 2:** Univariate analysis of factors influencing sustained clinical deterioration in knee OA patients.

Variables	Favorable prognosis group (*n* = 176)	Poor prognosis group (*n* = 65)	*t/χ^2^*	*P*
Age (years)	63.51 ± 6.94	64.23 ± 6.67	0.722	0.471
Gender (male/female)	80/96	30/35	0.009	0.923
BMI (kg/m^2^)	26.70 ± 3.90	29.90 ± 4.10	5.575	0.001
Symptom duration (months)	39.21 ± 17.85	42.82 ± 21.56	1.315	0.190
Prior injection frequency (times/year)	1.20 ± 0.60	1.30 ± 0.70	1.097	0.274
mJSW (mm)	3.22 ± 0.74	2.58 ± 0.72	6.002	0.001
IJSW (mm)	5.20 ± 1.05	4.97 ± 1.12	1.482	0.140
MOH (mm)	2.62 ± 0.68	2.81 ± 0.76	1.864	0.064
TBLV (cm^3^)	1.62 ± 0.82	2.26 ± 0.93	5.182	0.001
FMCCT (mm)	2.32 ± 0.46	2.19 ± 0.48	1.924	0.056
SBPT (mm)	3.54 ± 0.83	3.72 ± 0.88	1.470	0.143
MMED (mm)	3.78 ± 1.10	4.05 ± 1.15	1.671	0.096
TFA (degrees)	175.80 ± 3.20	178.50 ± 3.10	5.862	0.001
Pain VAS score (0–100 mm)	54.90 ± 13.10	58.20 ± 13.20	1.732	0.085
WOMAC pain subscore (0–20)	11.90 ± 3.30	11.50 ± 3.20	0.842	0.401
WOMAC function subscore (0–68)	37.50 ± 9.20	45.00 ± 10.30	5.435	0.001
6-minute walk distance (meters)	370.46 ± 70.53	351.25 ± 68.37	1.892	0.060
Active flexion angle (degrees)	123.59 ± 10.80	120.91 ± 10.92	1.705	0.090
Active extension lag (degrees)	2.61 ± 1.46	3.02 ± 1.61	1.881	0.061
Serum hs-CRP (mg/L)	3.32 ± 1.65	4.68 ± 2.10	5.259	0.001
uCTX-II (ng/mmol Cr)	328.50 ± 115.70	432.60 ± 128.20	6.018	0.001
30-second chair stand test (repetitions)	13.29 ± 3.51	12.63 ± 3.64	1.283	0.201

### Multivariate logistic regression analysis of factors associated with sustained clinical deterioration in knee OA patients

**Figure 1:**
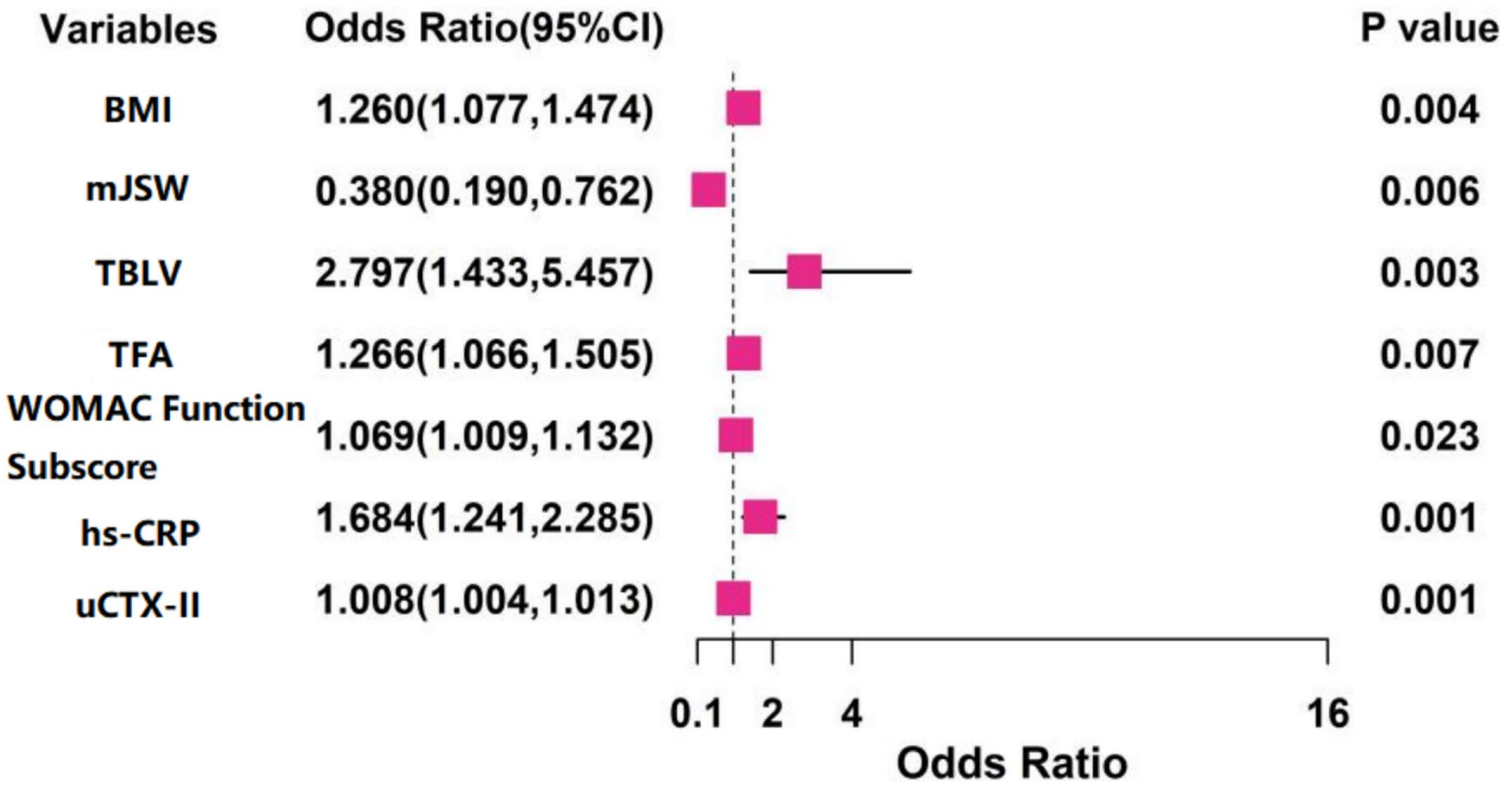
The results of multivariate logistic regression analysis.

Treatment outcome (favorable prognosis = 0, poor prognosis = 1) was designated as the dependent variable (Supplementary Table 1). Variables with statistical significance in univariate analysis were subjected to LASSO regression for feature selection, using the lambda.1se criterion (Supplementary Figures 1, 2). Selected predictors were incorporated into multivariate logistic regression, showing BMI (OR = 1.260, 95% CI:1.077–1.474, *p* = 0.004), TBLV (OR = 2.797, 95% CI:1.433–5.457, *p* = 0.003), TFA (OR = 1.266, 95% CI:1.066–1.505, *p* = 0.007), WOMAC function subscale score (OR = 1.069, 95% CI:1.009–1.132, *p* = 0.023), serum hs-CRP (OR = 1.684, 95% CI:1.241–2.285, *p* = 0.001), and uCTX-II (OR = 1.008, 95% CI:1.004–1.013, *p* = 0.001) as independent risk factors for sustained clinical deterioration, while mJSW (OR = 0.380, 95% CI:0.190–0.762, *p* = 0.006) was identified as an independent protective factor (Figure 1). In the regression model, the tolerance of each variable was >0.1, the VIF was <2, the condition index was < 30, and there was no situation where the variance proportion of multiple covariates under the same eigenvalue was > 50%. Therefore, there was no collinearity among the covariates.

### Machine learning model performance

**Figure 2:**
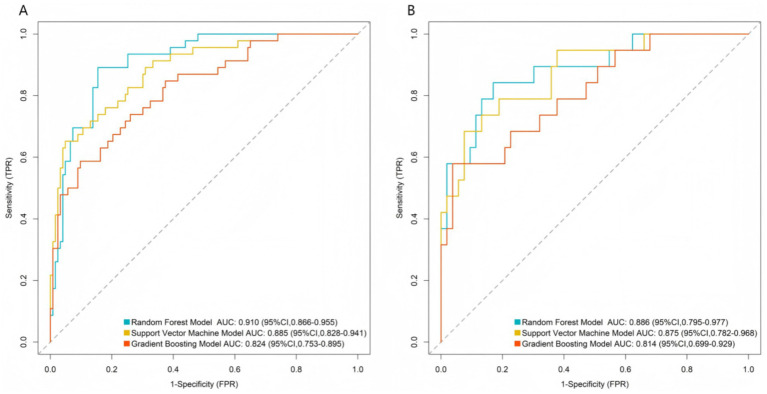
Receiver operating characteristic curves of machine learning models. **(A)** Training set; **(B)** validation set.

**Figure 3:**
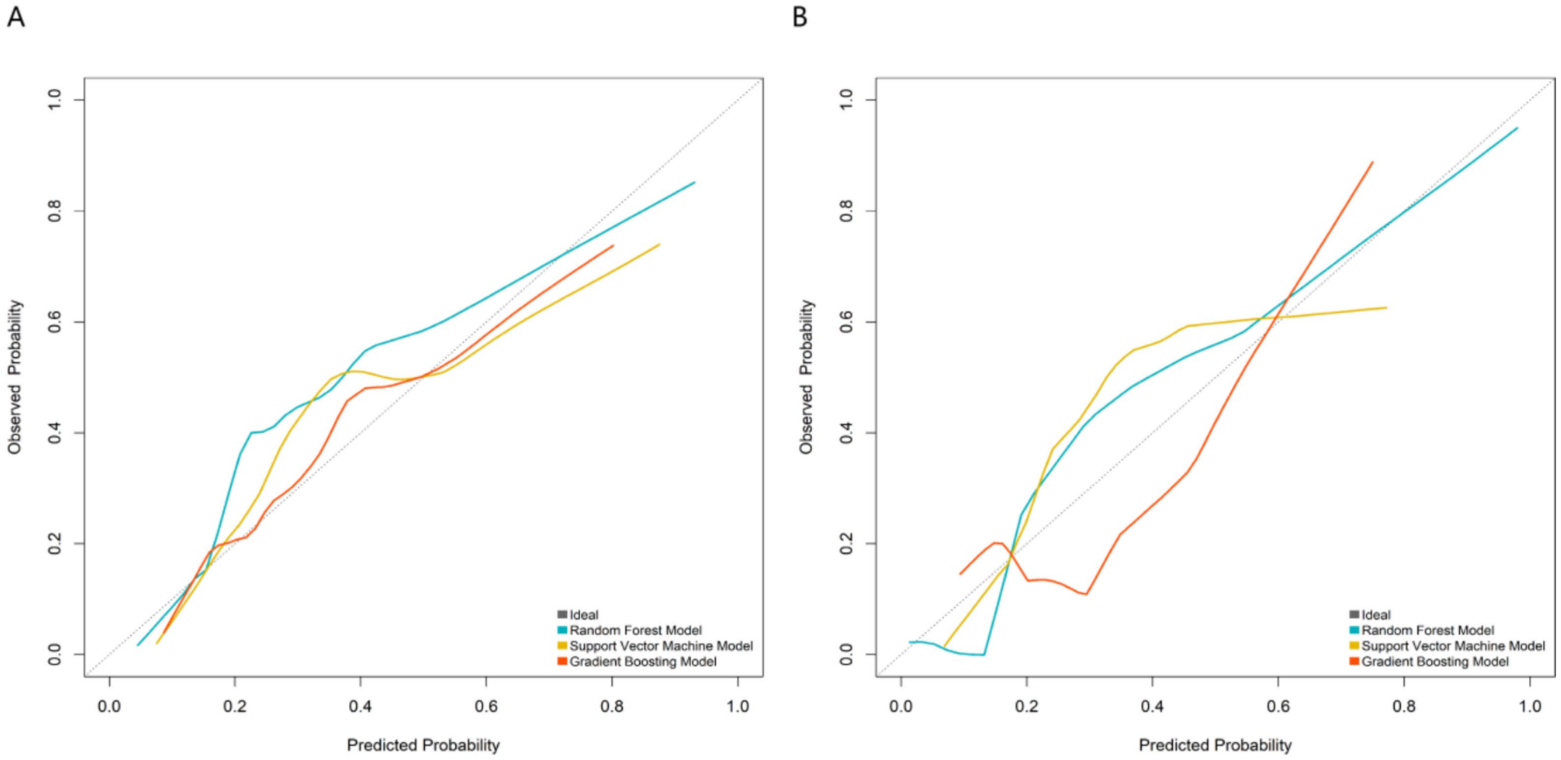
Calibration plots of machine learning models. **(A)** Training set; **(B)** Validation set. The closer the curve is to the 45° diagonal line, the better the calibration performance of the model.

**Figure 4:**
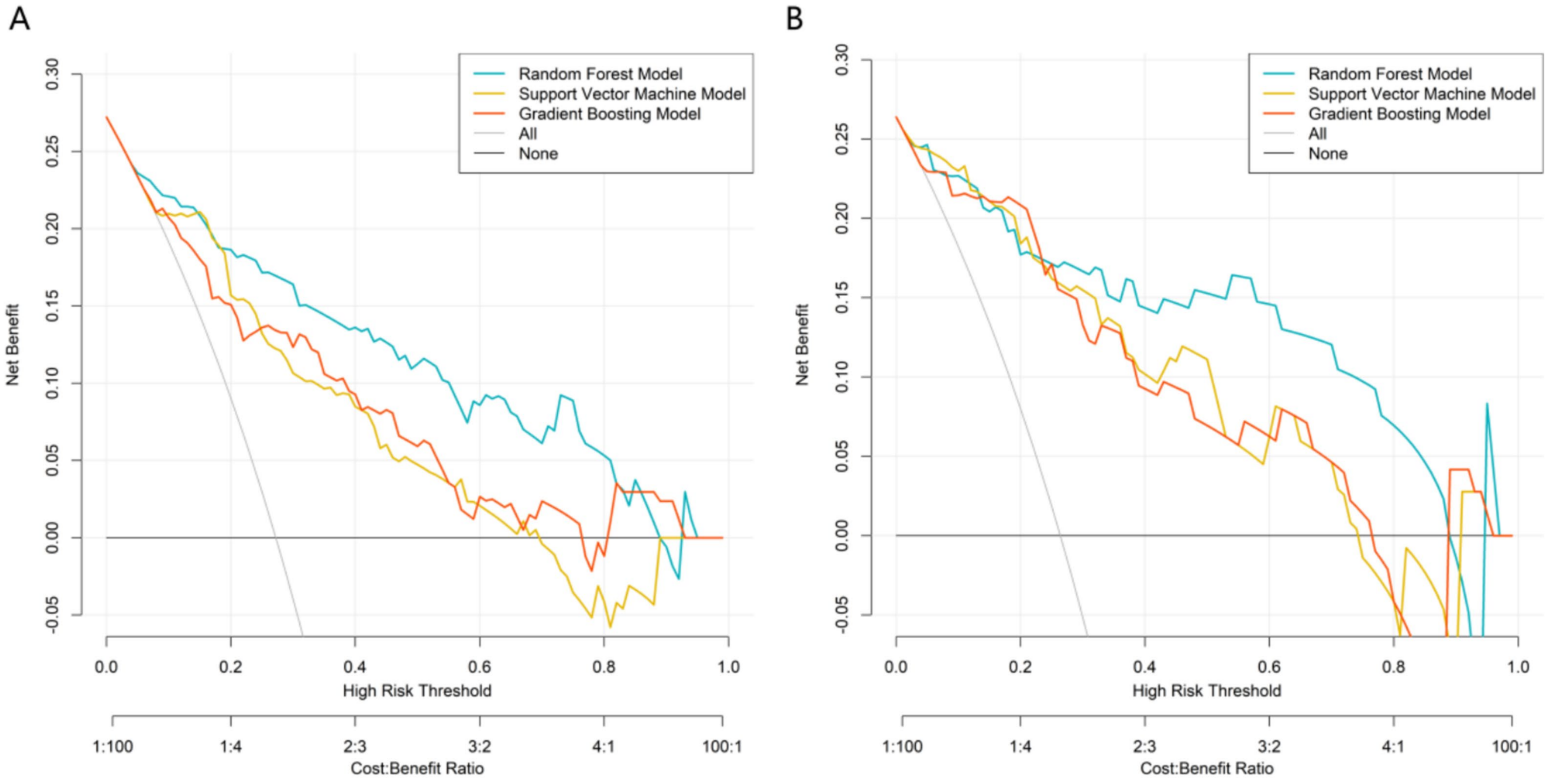
Decision curves of machine learning models. **(A)** Training set, **(B)** Validation set.

In the training set, the AUC values were 0.910 (95%CI 0.866–0.955) for RF, 0.885 (95%CI 0.828–0.941) for SVM, and 0.824 (95%CI 0.753–0.895) for GBM; in the validation set, the AUC values were 0.886 (95%CI 0.795–0.977) for RF, 0.875 (95%CI 0.782–0.968) for SVM, and 0.814 (95%CI 0.699–0.929) for GBM (Figure 2). Calibration evaluation was performed to assess the consistency between predicted probabilities and observed outcomes. Calibration plots showed that calibration curve RF model was closest to the ideal 45° diagonal line among the three models, indicating that its predicted probabilities were most consistent with actual clinical outcomes (Figure 3). DCA demonstrated that the RF model constructed based on multidimensional imaging features and clinical biomarkers consistently outperformed the extreme strategies of “full treatment” or “full non-treatment” in terms of clinical net benefit across a wide threshold probability range of 0.1–0.7, with its outstanding clinical utility further highlighted when compared to traditional prognostic assessment models (Figure 4). To sum up, the RF model was selected as the optimal predictive model.

### Predictive model construction

The RF model quantified variable importance scores for independent predictors of clinical deterioration. The descending order of importance was: BMI, TBLV, uCTX-II, hs-CRP, mJSW, TFA, and WOMAC function subscale scores (Supplementary Figures 3, 4). To further interpret the model, we calculated the SHAP values of each feature. The SHAP importance ranking was consistent with the variable importance score of the RF model, confirming BMI and TBLV as the top two key predictive factors.

**Figure 5:**
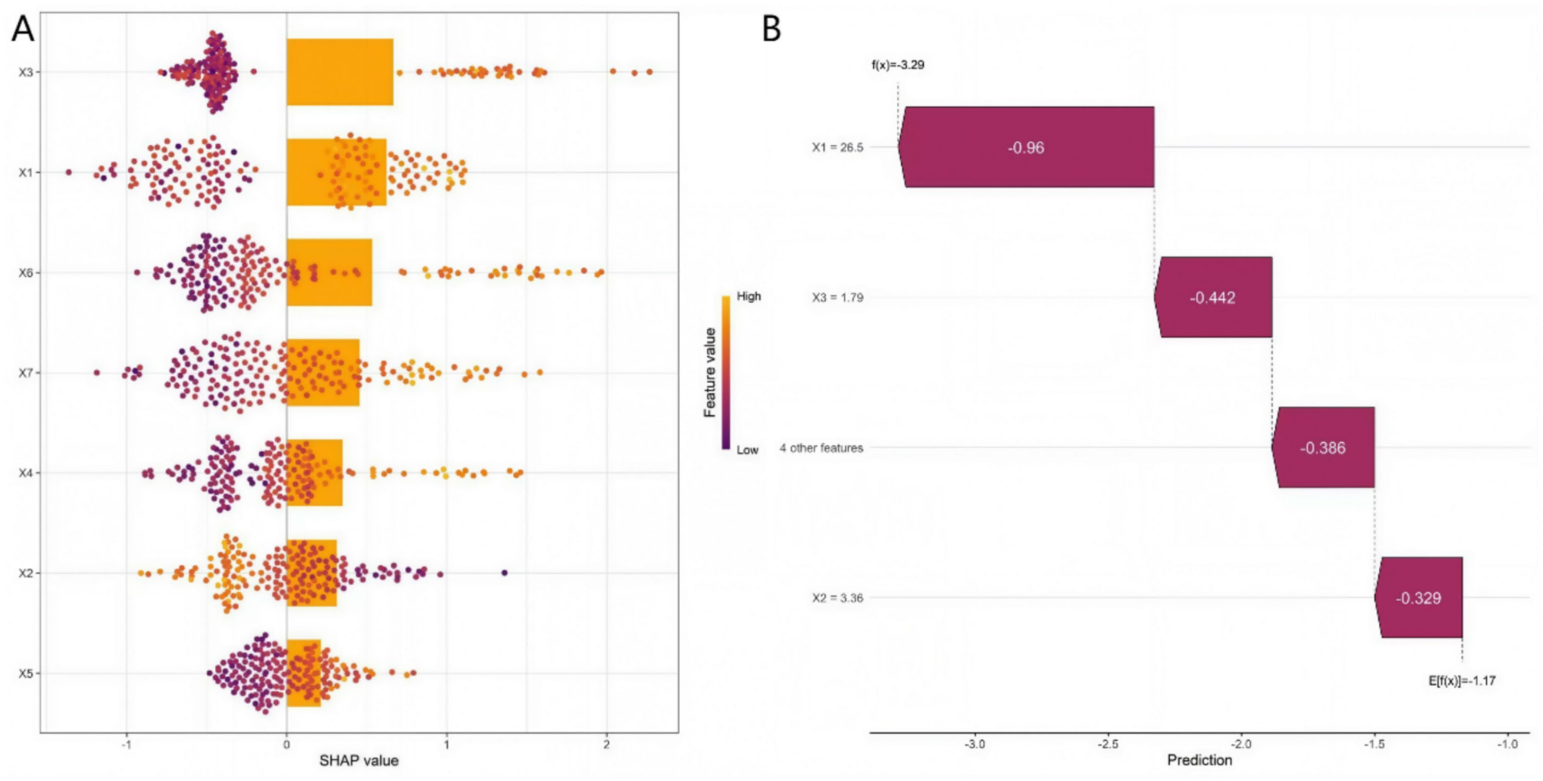
SHAP beeswarm plot **(A)** and SHAP waterfall plot **(B)** (X1: BMI, X2: mJSW, X3: TBLV, X4: TFA, X5: WOMAC function subscore, X6: hs-CRP, X7: uCTX-II).

**Figure 6:**
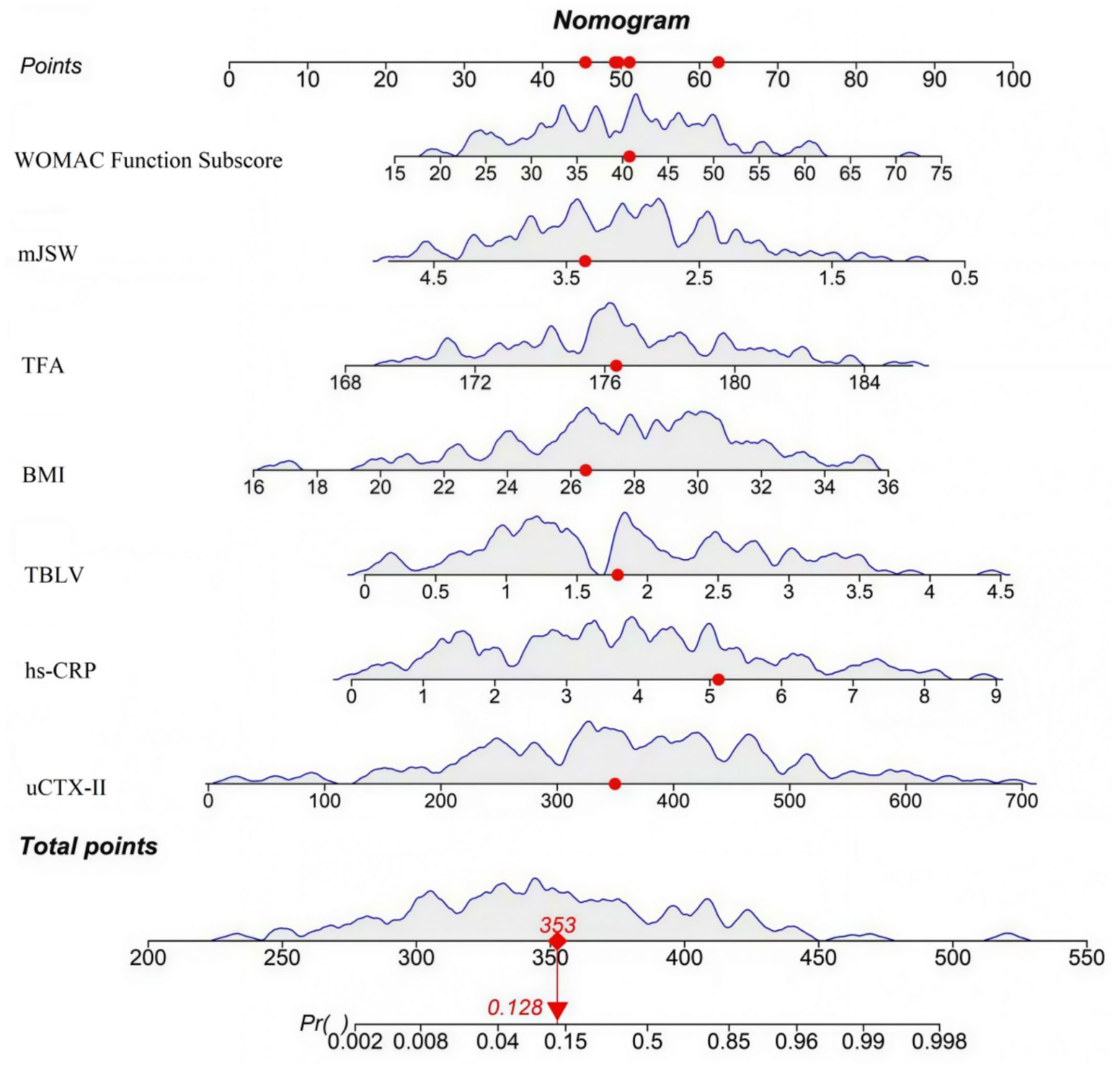
Fancy nomogram.

Figure 5A showed the distribution of SHAP values for each feature—positive values increase poor prognosis risk, negative values decrease it, reflecting each feature’s influence direction and magnitude. Figure 5B displayed a representative sample’s feature contributions, clarifying how each feature drives the prediction from the model’s average level. Figure 6 integrated key predictors: it quantifies feature weights (consistent with SHAP importance) and allows clinicians to calculate individual patient risk by summing feature scores, translating interpretability into clinical utility.

## Discussion

OA, as a common degenerative joint disease, exhibits significant heterogeneity in disease progression. Some patients rapidly progress to end-stage disease requiring joint replacement within a few years, while others remain clinically stable for extended periods (11, 12). Therefore, accurate early prediction of OA prognosis is crucial for implementing individualized interventions and optimizing healthcare resource allocation (13–15). In this study, we successfully developed and validated a comprehensive predictive model for OA using multidimensional data from 345 patients. Key independent predictors (BMI, mJSW, TBLV, TFA, WOMAC function subscale score, serum hs-CRP, and uCTX-II) were identified via rigorous statistical analysis, and the RF model outperformed SVM and gradient boosting with optimal predictive performance. The top-ranked feature importance of BMI, TBLV, and uCTX-II highlights that obesity severity, bone marrow lesion extent, and cartilage metabolism are core determinants of OA progression and treatment response. These findings provide scientific evidence for dynamic disease monitoring and personalized treatment optimization, with BMI’s significant influence further confirming the critical role of metabolic load in joint degeneration.

BMI emerged as a strong predictor of poor OA prognosis, acting through dual mechanisms: increased mechanical load accelerating cartilage wear, and adipokine-mediated chronic inflammation promoting cartilage degradation via matrix metalloproteinase activation (16, 17). This finding underscores the critical role of metabolic load in joint degeneration, consistent with previous evidence linking obesity to OA progression. The mJSW served as an independent protective factor against poor prognosis. As a direct surrogate for cartilage thickness, mJSW narrowing is a classic radiographic marker of OA progression. A wider baseline mJSW significantly reduced the risk of rapid disease progression or joint replacement, indicating that initial cartilage preservation plays a protective role in delaying OA worsening (18, 19). Additionally, an annual mJSW decline exceeding 0.2 mm was a sensitive indicator of rapid progression, suggesting that dynamic changes better predict structural damage than static measurements. Thus, monitoring baseline mJSW and decline rates is crucial for OA prognosis assessment, high-risk patient stratification, and clinical intervention guidance.

TBLV represents intraosseous abnormalities on MRI, reflecting micro-fractures, bone marrow edema, and fibrotic changes. Increased TBLV strongly correlated with poor outcomes, as it is not only a major source of pain but also a key driver of subchondral bone biomechanical deterioration and accelerated joint degeneration (20). TFA reflects lower limb malalignment, particularly varus deformity, which increases medial compartment loading. In this study, each 1-degree increase in TFA elevated the risk of poor prognosis by 12%. Serum hs-CRP, a systemic inflammation marker, was significantly associated with rapid OA progression. Persistent low-grade inflammation promotes synovial cytokine release, accelerating cartilage matrix degradation and synovitis (21). uCTX-II, a direct metabolite of type II collagen degradation, reflected rapid cartilage breakdown at levels >400 ng/mmol Cr (22). In this study, uCTX-II was a sensitive biomarker for radiographic progression, often preceding visible imaging changes. The WOMAC function subscale score, a core patient-reported outcome, was significantly linked to poor prognosis. Its deterioration reflects structural damage, pain, and stiffness, often driving surgical decision-making.

Although individual predictors such as BMI, mJSW, and uCTX-II have been previously identified as risk factors for OA progression, their combined predictive value in patients with standardized baseline treatment remains unclear. The novelty of this study lies in integrating imaging features, clinical scales, and biochemical biomarkers to construct a comprehensive prognostic model, which can quantitatively evaluate the synergistic effect of multiple predictors and provide a more accurate tool for clinical risk stratification.

This study had several advantages. First, by constructing multiple decision trees, RF effectively captured interactions (e.g., the synergistic worsening of osteoarthritis by BMI and inflammatory markers), which linear models (e.g., Logistic regression) often overlook. Second, Bootstrap sampling (out-of-bag error estimation) and random feature selection enhanced robustness. Third, RF performed well despite the variability in clinical data. Although the SVM and GBM showed slightly lower performance, their predictive capability remained clinically meaningful.

This study innovatively combined imaging, clinical scores, and biomarkers, overcoming single-data-source limitations and improving prediction accuracy. Rigorous cross-validation ensured model reliability, supporting future real-world applications. However, this study has some limitations. First, the single-center retrospective design and internal validation (random split within the same cohort) may lead to overestimation of model performance due to population homogeneity, and the reported AUC (0.910) may not reflect the actual performance in diverse clinical settings. Second, potential overfitting risk exists due to the relatively small sample size compared with high-dimensional features, although this was mitigated by the RF model’s anti-overfitting ability and 10-fold cross-validation. Third, radiomics features were not included, which may have limited the predictive performance of the model. Fourth, the study population was limited to knee OA patients with standardized baseline treatment, which may reduce the generalizability of the model to other OA populations. Future research should conduct multicenter, large-sample external validation (including temporal validation with different follow-up periods) to verify the model’s performance in diverse populations and improve its clinical applicability.

The clinical implications of this study are as follows: First, the RF model with high predictive accuracy can be used for early screening of high-risk KOA patients in clinical settings, enabling timely intervention to delay disease progression. Second, the key predictors (BMI, TBLV, uCTX-II) identified in the model can serve as targets for personalized treatment: for patients with high BMI, weight management should be emphasized; for those with increased TBLV, early intervention to reduce bone marrow inflammation may be beneficial; and for patients with elevated uCTX-II, drugs targeting cartilage degradation can be considered. For future research, multicenter external validation with larger sample sizes is needed to further improve the generalizability of the model. In addition, integrating radiomics features and omics data into the model may enhance its predictive performance. Moreover, longitudinal studies with longer follow-up periods are required to verify the long-term predictive value of the model.

In conclusion, this study developed and validated an integrated OA prognostic model. The RF model demonstrated superior performance, with key predictors including BMI, mJSW, TBLV, TFA, WOMAC function score, hs-CRP, and uCTX-II. This model facilitates early high-risk patient identification and personalized treatment strategies. Despite retrospective design limitations, multidimensional data integration and advanced machine learning significantly enhance OA prognosis prediction, advancing precision medicine in OA management.

## Data Availability

The original contributions presented in the study are included in the article/Supplementary material, further inquiries can be directed to the corresponding author.

## Glossary

OA: osteoarthritis
LASSO: least absolute shrinkage and selection operator
RF: Random Forest
KNN: K-Nearest Neighbors
SVM: Support Vector Machine
AUC: area under the curve
ROC: receiver operating characteristic
SHAP: SHapley Additive exPlanations
K-L: Kellgren-Lawrence
BMLs: bone marrow lesions
BMI: body mass index
hs-CRP: high-sensitivity C-reactive protein
uCTX-II: urinary C-terminal telopeptide of type II collagen
ML: Machine learning
WOMAC: Western Ontario and McMaster Universities Osteoarthritis Index
VAS: Visual Analogue Scale
KOOS: Knee injury and Osteoarthritis Outcome Score
SF-36: 36-Item Short Form Health Survey
HADS: Hospital Anxiety and Depression Scale
TUG: Timed Up and Go Test
30s-CST: 30-s Chair Stand Test
mJSW: Medial joint space width
lJSW: lateral joint space width
MOH: maximum osteophyte height
TBLV: total bone marrow lesion volume
FMCCT: femoral medial condyle cartilage thickness
SBPT: subchondral bone plate thickness
MMED: medial meniscus extrusion distance
TFA: tibiofemoral angle
OARSI: Osteoarthritis Research Society International
TKA: total knee arthroplasty
