# Supplementary material for: Prognostic model for osteoarthritis combining imaging and clinical biomarkers
Source: Front Med (Lausanne). 2026 Mar 18;13:1722232. doi: 10.3389/fmed.2026.1722232 (PMC13038948; doi:10.3389/fmed.2026.1722232)
Supplement: Supplementary file 1 [file Data_Sheet_1.docx]

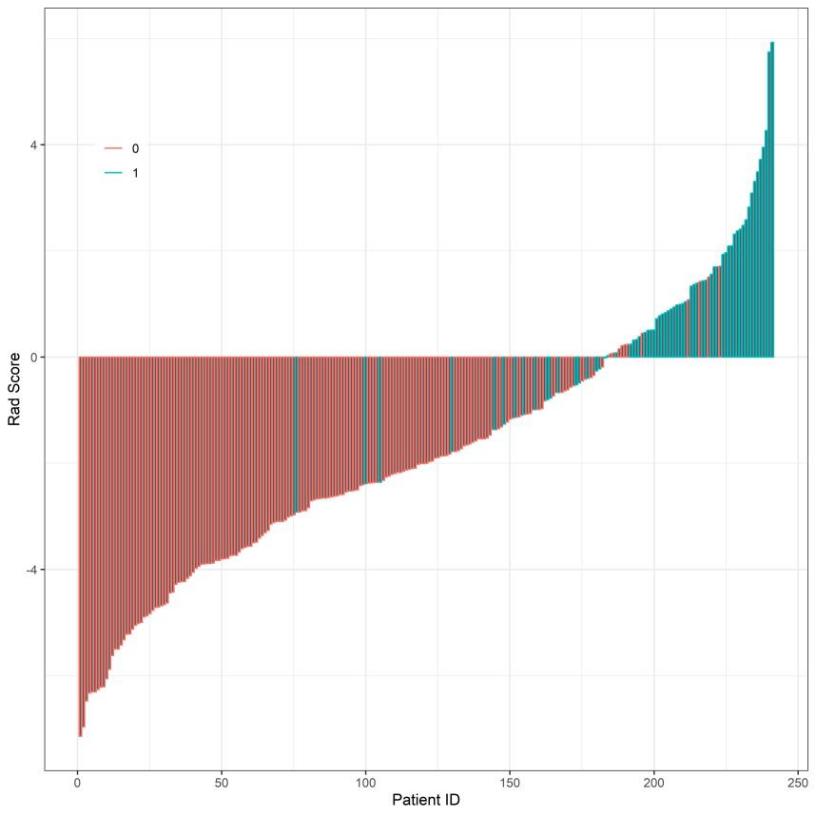


**Supplemental Figures 1.** LASSO coefficient profiles


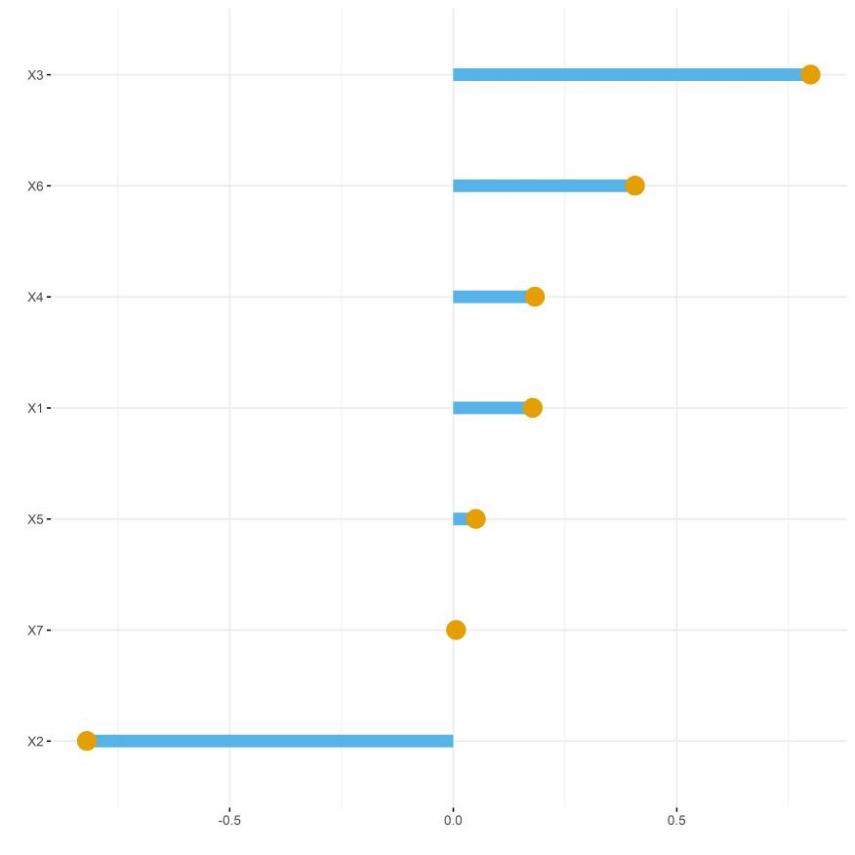


**Supplemental Figures 2.** Lambda.1se selection


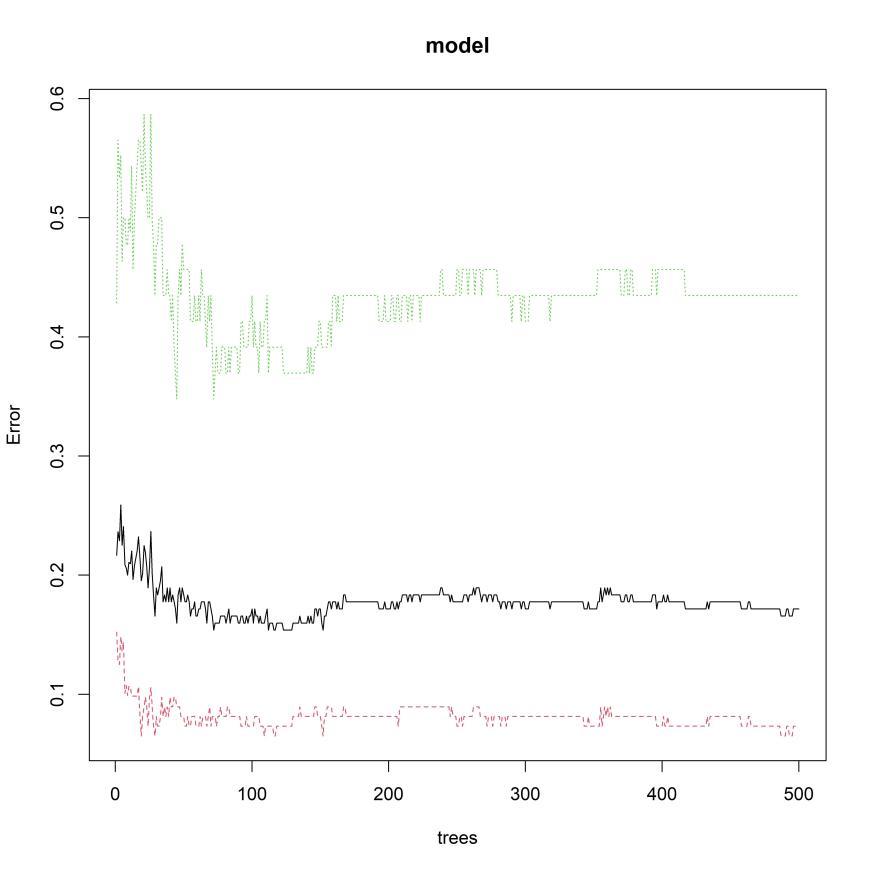


**Supplemental Figure 3.** Out-of-bag error rate versus decision tree number


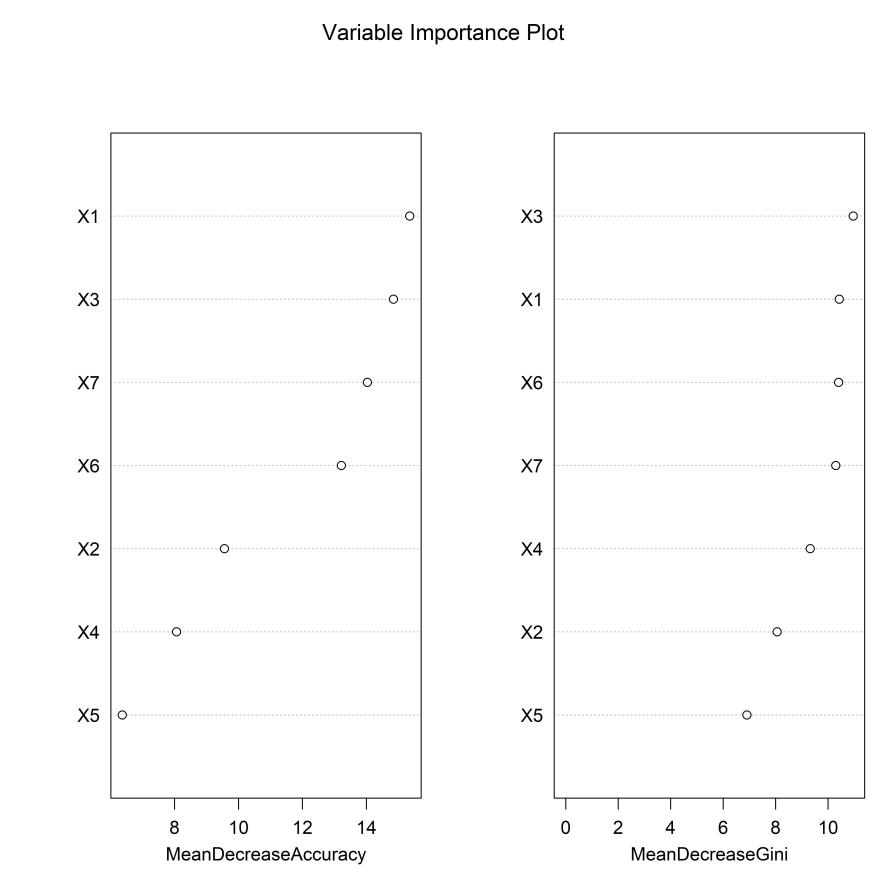


**Supplemental Figure 4.** Variable importance ranking in the RF model (X1: BMI, X2: mJSW, X3: TBLV, X4: TFA, X5: WOMAC Function Subscore, X6: hs-CRP, X7: uCTX-II).
