# Supplementary material for: Prognostic model for osteoarthritis combining imaging and clinical biomarkers
Source: Front Med (Lausanne). 2026 Mar 18;13:1722232. doi: 10.3389/fmed.2026.1722232 (PMC13038948; doi:10.3389/fmed.2026.1722232)
Supplement: Supplementary file 2 [file Table_1.docx]

**Supplemental Table 1.** Variable assignments

| Variable | Meaning | Assignment |
| --- | --- | --- |
| X1 | BMI | Continuous |
| X2 | mJSW | Continuous |
| X3 | TBLV | Continuous |
| X4 | TFA | Continuous |
| X5 | WOMAC Function Subscore | Continuous |
| X6 | Serum hs-CRP | Continuous |
| X7 | uCTX-II | Continuous |
| Y | Clinical deterioration | Favorable=0, poor prognosis=1 |
